# Resilience and Higher Education Support as Protective Factors for Student Academic Stress and Depression During Covid-19 in the Netherlands

**DOI:** 10.3389/fpubh.2021.737223

**Published:** 2021-10-21

**Authors:** Melissa Versteeg, Rutger Kappe

**Affiliations:** Department of Student Success, InHolland University of Applied Sciences, Haarlem, Netherlands

**Keywords:** academic stress, Covid-19, depression, higher education, resilience, wellbeing, students, support

## Abstract

**Background:** The corona pandemic has forced higher education (HE) institutes to transition to online learning, with subsequent implications for student wellbeing.

**Aims:** This study explored influences on student wellbeing throughout the first wave of the corona crisis in the Netherlands by testing serial mediation models of the relationships between perceived academic stress, depression, resilience, and HE support.

**Methods:** The Covid-19 International Student Wellbeing Study (C19 ISWS) was used, with a total sample of 2,480 higher education students studying at InHolland Universities of Applied Sciences in the Netherlands. Student subgroups were created, so that students with low and high perceived academic stress could be assessed, in addition to depressed and non-depressed students. Predictive model fit was tested using Macro PROCESS.

**Results:** A significant serial mediation model for the total student sample was revealed, including protective mediating effects of resilience and HE support on the positive direct effect of perceived academic stress on depression. At subgroup level, significant (partial) predictive effects of resilience on depression scores were noted. A partial serial effect between resilience and HE support was found for students with low perceived stress levels, whereas a parallel partial mediation model was present among highly academically stressed students. Regarding non-depressed students, a full parallel mediation model was found, whereas the model for depressed students inadequately explained the data.

**Conclusions:** Overall, resilience and HE support mediate the predictive effect of academic stress on depressive symptoms among students. In addition, substantial differences in model fit arise when inspecting the students on a subgroup level. These findings contribute to the gap in knowledge regarding student wellbeing during the Covid-19 pandemic in the Netherlands, in addition to providing novel insights on student subgroup dynamics. While Covid-19 restrictions continue to demand online learning, student wellbeing may be enhanced overall by targeting resilience and increasing awareness and availability of HE support services. The current study also highlights the need for differential approaches when examining wellbeing for specific student groups.

## Introduction

Higher education (HE) students face profound lifestyle changes. Moving away from home, changes in peer support, an increase in decisions regarding personal and professional Opportunities, and elevated levels of stress from relationships can interfere with wellbeing (1–5). During student life, over half of enrolled students experience emotional problems (3) which may result from academic overload, pressure to succeed, peer competition, and less time to spend on leisure or family (6). When students experience psychological distress, academic productivity drops (7). Students who are experiencing depression or other psychological problems are generally found to have trouble maintaining progress, and encounter difficulty adjusting to higher education (3). Academic failure rises with increased psychological problems in students and many students report psychosocial issues prior to dropping out (7, 8).

For 2019 and 2020, Dutch populations aged 18 to 25, reported the highest levels of unhappiness compared to other age groups, in addition to reporting the highest levels of dissatisfaction regarding the state of their mental health (9). In the Netherlands, major depression has been identified as the most common individual mental health disorder (10). Global analyses indicate that psychological disorders will have presented by age 24 in 75% of cases (11). With major depressive disorders, occurrence during earlier life stages increases recurrence rates during early adulthood by 400% (12). With an average age at higher educational graduation of 23.4 years for students in the Netherlands (13), a substantial group will experience psychological problems during student life. The bulk of such problems are predicted to occur among younger students and estimates suggest the prevalence of psychological problems to be over 30% among student populations (3, 7, 14).

Besides psychological problems such as depression, stress is also reported to play a major role in student wellbeing (15–17). Greater perceived academic stress, including increases in perceived academic workload, and higher levels of loneliness within the academic context, reveal a stronger effect on depression than do indicators of cumulative academic demands or academic grades (18). In the Netherlands, an increasingly demanding student life has been reported. Performance pressure, finances, and rigid study continuation criteria are noted as important academic contributors to stress among Dutch students (7). Moreover, students' stress appraisal directly impacts development of psychological problems. For those who feel that stress is negative, and perceive it as involving serious consequences or threat, frequent stress exposure is linked to higher levels of psychological distress and use of support services (16, 19, 20).

To help students effectively cope with stress and mental health issues, support resources are required (21, 22), and studies indicate a potential role for the HE institutes in providing these resources (23–25). In the presence of adequate support, the effect of stress on development of psychological problems is reduced and may even be fully remedied (26). However, for younger people, experiencing psychological distress is often accompanied by perceived stigma, feelings of embarrassment, and a preference for self-reliance, which hamper formal help-seeking behaviours (24). In addition, students with depression or anxiety who fail to seek support frequently report unawareness of available services (27). Students with higher levels of distress are less able to effectively seek help. This highlights the possibility for educational institutes to increase education, awareness, and availability of wellbeing promoting facilities, including online resources (24, 25).

Student wellbeing during the coronavirus (Covid-19) outbreak is heavily impacted, with students reporting higher levels of hardship and vulnerability during this global health crisis. Students, as compared to other population groups, experience added duress due to the educational transition to a predominantly online environment (5, 28–30), and report significant stress caused by changes in teaching methods (31). Grappling with the transition to remote learning presents challenges to students, with the effects of the pandemic described as removing “both the opportunity and the will to be productive” (32). Government-imposed restrictions require social distancing and isolation, with subsequent increases in psychological distress and development of disorders including stress, depression, irritability, and insomnia (33). These emerging threats are inciting higher educational institutes to prioritise questions concerning their duty of care for student wellbeing (34), which motivated investigation of HE support facilities in the current study.

Trends of elevated psychological susceptibility during Covid-19 are found globally among student populations, including students in China, Italy, England, Greece, the United States, Germany, and France (5, 28, 35–42), but have yet to be studied in the Netherlands. The observed declines in wellbeing are attributed to online learning, isolation from peers, strained relationships with teachers and classmates, and relocation following school campus closure (5, 43). A recent meta-analysis estimated the overall prevalence of depression among students to range from 32.9 to 49.1% (44).

The first recorded case of the coronavirus in the Netherlands occurred during early 2020. Following its rapid spread, the government announced restrictions with stringency increases as the Covid-19 virus spread (Table A1). Among these restrictions, a call to stop teaching at location was announced. An expansion of government-imposed restrictions continued until May 2020, after which a first tentative step towards limited reopening was introduced, as the first wave of infections receded (45, 46).

Examining students' perspectives on how educational institutes may enhance wellbeing reveals relevant themes for HE student services and support facilities during the Covid-19 restrictions. From students' standpoint, increasing awareness of services, promoting their use, and improving availability, range, and quality of support services is instrumental to increased wellbeing (23). With current restrictions demanding an expansion of online facilities, HE services may seek to expand online support facilities. Reviews of web-based and computer-delivered interventions describe benefits to student mental health, with around 50% demonstrating at least one significant positive outcome following online interventions (47–49).

Resilience is also a much-favoured way to promote student wellbeing, through its positive impact on one's capacity to effectively navigate stressful environments (1). Resilience, defined as the ability to bounce back following stress exposure (50), is portrayed as a vital component to adaptive recovery, and refers to stabilisation following threats to wellbeing (51). In a meta-analysis, it was shown that resilience is positively correlated with indicators of mental health (52). More so, the positive relationship between resilience and mental health is strongest following exposure to significant adversity. Thus, to effectively activate resilience, stress exposure is required (52–54).

Resilience is pivotal to maintaining balance on individual and societal levels and is especially relevant during the Covid-19 pandemic (55). Covid-19 studies on wellbeing emphasise resilience as mediating the negative outcomes related to Covid-19 stress and fears (56, 57). According to the challenge model of resilience (58), an optimal range of stress exposure exists within which individuals can cultivate resilient response. Stress levels that are too low activate sub-optimal resilient responses, and stress levels that are too high predict negative outcomes as stress exposure becomes overwhelming. In addition, strong ties exist between resilience and support, where support promotes resilient recovery following stress exposure, in addition to improving help-seeking attitudes and increasing one's capacity to identify and utilise supportive resources (24, 59, 60).

With evident roles for academic stress, resilience, and support on student wellbeing, the current study proposes an explanatory model to explain the relationships between Covid-19 related academic stress, depression, resilience, and HE support for students studying at HE institutes throughout the Netherlands during the coronavirus pandemic. The Dutch HE system involves two distinct forms of higher education. The first regards academic research oriented higher education, offered by universities (in Dutch: wetenschappelijk onderwijs). The second form includes higher professional education offered by universities of applied science (in Dutch: hoger beroepsonderwijs). The current study included higher professional education students to ensure sample homogeneity.

**Figure 1:**
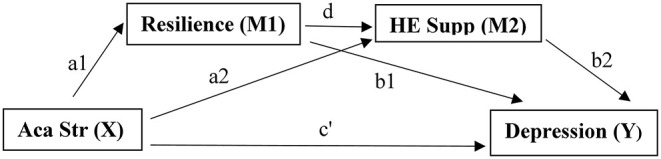
Explanatory serial mediation model with hypothesised direct pathway, independent mediation pathways, and serial mediation pathway between academic stress, depression, resilience, and HE support. Aca Str, academic stress; HE Supp, higher education support.

The hypothesised model of academic stress on depression during Covid-19 used in the current study includes a direct predictive effect of academic stress perception on reported levels of depression, in addition to proposing three predictive indirect effects: (1) a partial mediation effect of resilience, where higher resilience has a stronger protective effect on depression, (2) a partial mediation effect of HE support, where students who report higher identification of support facilities available within the HE context are believed to also experience a stronger protective effect on depression rating, and (3) a partial serial mediation effect where the indirect effect of resilience and HE support in succession offers a protective effect on depression in HE students (see Figure 1).

Previous studies indicate a need to focus on differential effects between student subgroups to address gaps in available knowledge (7, 18, 57). As such, the current study included model fit tests for student subgroups. Predictive model fit examinations were carried out for all eligible HE students studying in the Netherlands, and model fit was subsequently analysed for four students subgroups: (1) HE students who report low perceived academic stress during the Covid-19 pandemic, (2) HE students who report high perceived academic stress during the Covid-19 pandemic, (3) HE students whose reported levels of depression approaching clinical diagnoses of depression (61), and (4) HE students whose depressive symptom profiles are below the threshold for clinical depression. Based on available literature (57, 58), we expect that higher levels of stress decrease the protective mechanisms provided by resilience and the ability to identify HE support. In addition, we expect that students reporting depressive levels linked to clinical depression will experience a lower protective effect of resilience and HE support. The current study thus provides a novel model analysis of student wellbeing throughout the Covid-19 pandemic in the Netherlands, and additionally includes novel examinations of wellbeing among student subgroups.

## Method

### Survey

Across 26 countries and 110 higher educational institutes, students were invited to complete the Covid-19 International Student Well-being Study (C19 ISWS). Invitations were sent via email, where participating research groups received a country-specific, or institute-specific link for survey distribution. This approach limited survey distribution and subsequent data collection to respondents studying in the Netherlands (29). The survey was completed online *via* Qualtrics survey tool in accordance with European guidelines on General Data Protection Regulation (GDPR). The study was approved on ethical standards as defined by the Ethics Committee for Social Sciences and Humanities at the University of Antwerp, in addition to meeting standards set by the institutional review board at the InHolland University of Applied Sciences.

The C19 ISWS includes seven domains: (1) sociodemographic information, such as age, gender, and migration status, (2) study-related information such as study field, HE institute, and perceived importance of study, (3) changes due to the Covid-19 outbreak, including financial resources, living conditions, lifestyle, and activity levels, (4) Covid-19 infections, symptoms, and concerns like comorbidity, stigma, and risk perception, (5) stressors, informal support, and mental wellbeing, (6) student-specific questions and concerns such as help-seeking behaviours, perceived stress, and satisfaction with the HE communication strategies, and (7) Covid-19 knowledge and information, including questions on the students' attitudes towards government-imposed restrictions and communication strategies. The C19 ISWS questionnaire is available elsewhere (62).

The C19 ISWS was designed to measure a broad range of theme's including several widely implemented scales to collect data on wellbeing just prior to, or after, the initial peak in Covid-19 infections. In the Netherlands, participants of the C19 ISWS completed the survey between May 6th 2020 and May 18th 2020. The Dutch government reduced some of the lockdown restrictions following several weeks of an “Intelligent Lockdown” phase on May 11th, 2020, which included a reopening of middle schools, but this ease of restrictions included no changes relevant to teaching methods at HE institutes (see Table A1).

### Participants

All study participants were HE students, actively enrolled in a study programme at InHolland Universities of Applied Sciences throughout the Netherlands. Participation was voluntary and participants were invited to partake in the study if they were currently enrolled and were aged 17 years or older. Participants were required to give consent prior to proceeding. Of the participants who partook in the study, those who had successfully completed the survey were included in the dataset.

The cohort of students included 2,835 participants. Data homogeneity inspection revealed that enrolment status created a significant impact on the distribution of the dependent variable; *F*(3, 2,821) = 15.760, *p* < 0.001. *Post-hoc* contrasts indicated that the effect of fulltime enrolment was significantly different from other forms of enrolment. Furthermore, students who identified as gender “x” included 10 individuals who scored significantly higher on dependent variable [mean (M) = 2.675, standard deviation (SD) = 0.753] than students who identified as male (M = 2.193, SD = 0.630) or female (M = 2.273, SD = 0.611) (*t*(827) = −2.399, *p* = 0.009; *t*(2,104) = −2.071, *p* = 0.019). As previous examination of the CES-D8 has not validated reliable use for this gender group (63), gender “x” was excluded from the final dataset. Subsequently, fulltime HE enrollers, and those identifying as male or female were included in the final dataset.

The final sample used for analysis included 2,480 students with fulltime, 4 year HE enrolment of mean age 21.78 years (SD = 3.155). Of the respondents 775 were male (30.4%) and the remaining 1,725 were female (69.6%). The study sample is described in further detail in Table 1.

**Table 1:** Sociodemographic characteristics of participants (*N* = 2,480).

	***N***	***%***
**AGE IN YEARS**		
<18	260	10.5
18–20	678	27.3
21–23	995	40.1
24–27	426	17.2
>28	121	4.9
**GENDER**		
Male	755	30.4
Female	1,725	69.6
**STUDY YEAR**		
First	739	29.8
Second	594	24.0
Third	497	20.0
Fourth	453	18.3
Fifth or more	197	7.9
**MIGRATION STATUS**		
Born inside the Netherlands	2,219	89.5
Born outside the Netherlands	261	10.5
**PARENTAL EDUCATION**		
**Father**		
Less than secondary	132	5.3
Secondary	1,115	45.0
Higher education	1,044	42.1
Unknown	189	7.6
**Mother**		
Less than secondary	111	4.5
Secondary	1,276	51.5
Higher education	957	38.6
Unknown	136	5.5
**HIGHER EDUCATION GENERATION**		
First generation	1,075	43.3
Other	1,405	56.7

### Measurements

#### Depression

The level of depressive symptoms was measured with the Centre for Epidemiological Studies-Depression scale (CES-D8) short version, which was integrated in the C19 ISWS and has been tested as a reliable and valid tool to measure depressive symptoms in men and women (63, 64). This 8-item questionnaire asks respondents to indicate to which degree they agree with statements that reflect on thoughts, feelings, emotions, and energy levels over the past week. Responses are given according to a 4-point Likert scale, where 1 indicates “none or almost none of the time”, 2 indicates “ some of the time”, 3 indicates “most of the time”, and 4 indicates “all or almost all of the time”. The items ask an estimation of “how much of the time during the last week…” followed by specific item content, such as “you felt everything was an effort”, “your sleep was restless”, or “you felt sad”. The total scores are averaged, with higher scores indicating a higher presence of depressive symptoms, and a summed mean of 3.0 or higher indicating depression (61, 65, 66). Cronbach's alpha for the CES-D8 in the sample was 0.860. The sample had an average CES-D8 score of 2.280, with a standard deviation of 0.619. When grouped into a depressed and non-depressed subset according to scoring procedures, 16.3% reported symptom levels in accordance with clinical depression (61) (*N* = 404).

#### Academic Stress

Covid-19 related academic stress was measured using a 4-item scale included in the C19 ISWS domain on student-specific questions and concerns within the specific context of the coronavirus pandemic. All four items enquired about perception of changes in academic stress following the coronavirus pandemic and changes in students' academic experiences. Factor analyses conducted by the C19 ISWS consortium revealed four items which adequately assessed perceived academic stress during the transition to online teaching (29). This short assessment included evaluation of academic workload, course expectations, completion of the academic year, and teaching methods (63). Corresponding items included statements such as “my university/college workload has significantly increased since the Covid-19 outbreak” and “I am concerned that I will not be able to successfully complete the academic year due to the Covid-19 outbreak”. Responses were required along a 5-point Likert scale were 1 indicated “total agreement”, 3 indicated a “neutral” response, and 5 indicated “total disagreement” with the statement. All four item scores had to be reversed to allow intuitive interpretation, and a summed mean score was calculated. As such, a higher mean score indicates higher perceived academic stress following the Covid-19 restrictions and the HE transition to online teaching, with total scores ranging from 4.00 to 20.00, and summed mean scores between 1.00 and 5.00. A reliability analysis on the current dataset resulted in a Cronbach's alpha of 0.730. The respondents scored an average of 3.371 on the summed mean academic stress scale, with a standard deviation of 0.854.

In addition, academic stress scale total scores (range 4.00–20.00) were transformed to subset two groups; those who experienced low levels of academic stress, and those who experienced high levels of academic stress. The average total score on the academic stress scale for the sample was 14.86 (SD = 3.41). Cut-off scores were based on the total scores to allow clean cut-off lines inherent to the use of Likert-scale responses. As such, students scoring between the minimum +1 SD (4.00–7.41) were assigned to the low academic stress group, and the maximum score −1 SD (16.59–20.00) was used to identify the high academic stress group. In practise, as the academic stress scale is based on a 5-point Likert response scale, the low academic stress group scored between 4.00 and 7.00 (*N* = 63, M = 1.480, SD = 0.289) whereas the high academic stress group scored between 17.00 and 20.00 (*N* = 839, M = 4.611, SD = 0.282). Following group allocation, the summed mean scores of the academic stress scale were used for subsequent analyses.

#### Resilience

Resilience was measured using the Brief Resilience Scale (BRS) which is a short, self-reported 6-item measure of resilience with proven validity and reliability in other cohort studies (50). This scale was included in the C19 ISWS within the country-specific module. An indication of agreement with the statements was required according to a 5-point Likert scale. 1 indicated “total disagreement”, 3 indicated a neutral response, and 5 indicated “total agreement” with the provided statements. BRS items included “I tend to bounce back quickly after hard times”, “I have a hard time making it through stressful events”, and “it does not take me long to recover from a stressful event”. Three of the items had to be reversed prior to summing and averaging scores. Within the sample, the reliability analysis of the BRS revealed a Cronbach's alpha of.840. The respondents scored an average on the BRS of 2.952 with a standard deviation of 0.755. Scale summed scores can be grouped to classify resilience levels (65). BRS summed total scores between 1.00 and 2.99 are categorised as low resilience, 3.00–4.30 as normal resilience, and 4.31–5.00 as high resilience. Within the sample, 46,8% (*N* = 1,160) could be classified as having low resilience, 49.7% (*N* = 1,233) were classified as having normal resilience, and the remaining 3.5% (*N* = 87) had a high level of resilience. Using these group norms, the average resilience level of the final sample could be classified as “low”.

#### HE Support

Definitions of resilience include resilient behaviours through the identification and utilisation of supportive resources (59). The C19 ISWS item assessing students' identification and satisfaction with support facilities; “There are sufficient support facilities within the HE institute (e.g., student counselling, online support)” was included in the model. Responses were required on a 5-point Likert scale. Scores were transformed so that higher scores indicated higher perceived availability of supporting facilities within the HE setting. For the sample, 27.6% indicated strong disagreement, or disagreement with the statement (*N* = 156, *N* = 528, respectively), whereas 26,5% either agreed or strongly agreed (*N* = 595, *N* = 61, respectively). The remainder of the sample maintained a neutral attitude towards the availability of sufficient support facilities at the HE institute (*N* = 1,140).

### Statistical Analysis

IBM SPSS Statistics for Windows, Version 27.0 was used to carry out statistical analyses. The extension Macro PROCESS (67) version 3.5 was used to test model fit regarding a serial mediation effect by resilience and HE support on academic stress and depression. To estimate power probabilities for the subgroups examined for model estimation differences, G^*^Power software version 3.1.9.6 was used (68).

The serial mediation analysis was run with Macro PROCESS to estimate effect sizes and model fit for five groups: (1) all HE students, (2) students who report experiencing low levels of academic stress during the Covid-19 pandemic, (3) students who report high levels of academic stress during the Covid-19 pandemic, (4) students with CES-D8 scores indicating the presence of depression, and (5) students whose CES-D8 scale mean indicated the absence of depression.

During each group analysis, the nature of the relationship between X and Y (X: academic stress and Y: depression levels) was assessed directly, in addition to testing the indirect effect resulting from the two mediators resilience (M1) and HE support (M2), and their indirect serial mediation effect (Figure 1). The analytical workflow was based on previous work by Preacher and Hayes (69) where multiple mediation analysis is based on two elements. First, an examination is made to conclude whether the set of mediators transmits the effect of X to Y, and second, the specific indirect effect associated with each presumed mediator is tested. Within this framework, total indirect effects need not be significant for identification of relevant specific indirect effects.

Total, direct, indirect, and partial effects included in the model were described as statistically significant if the corresponding 95% confidence interval of the unstandardised effect size coefficient *b* did not contain zero. If the direct path between X and Y (c′) was significant, and all three indirect pathways (a1 x b1; a2 x b2; and a1 x d x b2) yielded significant results, a partial serial mediation model is present. If the c′ path effect between X and Y is non-significant and the three indirect pathways were significant, a full serial mediation model is present. If any of the indirect pathways fail to reach significance, the remaining indirect pathways were examined to assess the model.

During the Macro PROCESS analyses, bootstrap resampling value was set at 5,000. Each of the pathways was tested by regressing the corresponding variables. If the *b* coefficient of the estimated direct, serial indirect, or independent indirect effects occurred within a 95% confidence interval range excluding zero, the null hypothesis of no significant predictive effect was rejected.

No missing data was present for the sample as only completed surveys were included. More so, due to the Likert-scale response methods employed to measure all included variables within the hypothesised model, no outliers were identified. The final dataset (*N* = 2,480) was screened for violations that would prevent accurate use of Macro PROCESS. Although normality testing revealed non-normal data (Shapiro-Wilk statistic = 0.76, *p* < 0.001), bootstrapping techniques used in PROCESS are robust against violations of normality by using confidence intervals to assess effect significance (69, 70). *Post-hoc* examination of power revealed that groups had sufficient detection power. All HE students, high academic stress students, and non-depressed students maintained a power coefficient of 1.000. A power coefficient of 0.999 was found for low academic stress students, and the power coefficient was 0.971 for depressed students (68).

Assessments were run to determine the presence of covariates. A two-step approach was used to examine sociodemographic variables for linear effects on depression. First, based on literature (3, 5, 35, 38, 71), age, gender, migration background (“where you born in the Netherlands, or outside of the Netherlands?”), and family educational background of students (first generation HE student, vs. not the first generation) were selected and tested for significant effects on depression. During the second step, tests were run to determine each sociodemographic variable's relation to the independent variables academic stress, resilience, and HE support (72). All sociodemographic variables significantly correlated to at least one of the independent variables. Therefore, none were selected as covariates (see Table A2).

C19 ISWS data collection dates collided with government-induced changes in Covid-19 lockdown restrictions in the Netherlands. The survey was completed from May 6th 2020 until May 18th 2020, with a mean completion date of May 9th 2020 and a standard deviation of 3 days. As the introduction of the first steps towards reopening were introduced on May 11th, the dataset was inspected to cheque for date dependent effects on depression or academic stress ratings. Statistical analysis yielded no significant results (*p* = 0.089 and *p* = 0.194, respectively).

## Results

### All Students

**Figure 2:**
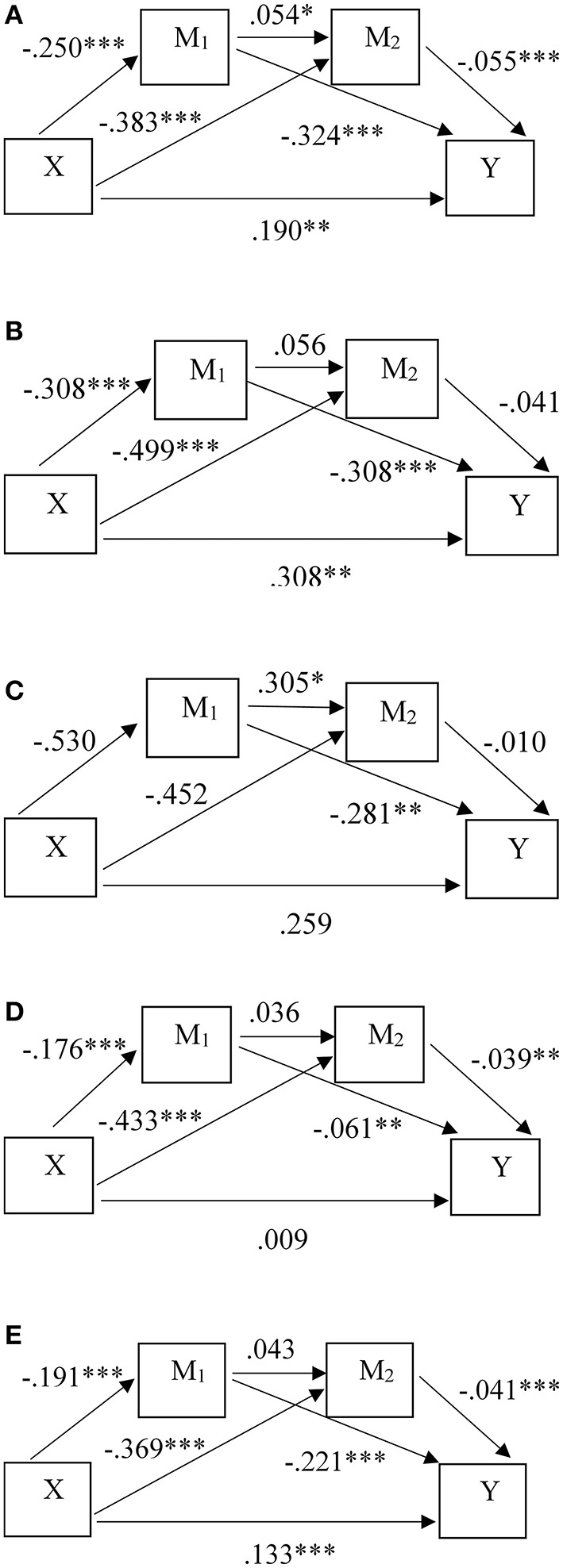
Predictive model effects for the HE student groups: **(A)** all students (*N* = 2,480), **(B)** low academic stress students (*N* = 63), **(C)** high academic stress students (*N* = 839), **(D)** depressed students (*N* = 404), **(E)** non-depressed students (*N* = 2,076). *significant at *p* < *0.05*, **significant at *p* < *0.01*, ***significant at p < 0.001.

For all students, the total predictive effect of the model was 0.293 (see Tables 2, **4**, Figure 2A). 64.85% of the effect originated in a direct effect between perceived academic stress and depressive symptoms, where higher levels of perceived academic stress significantly predict higher levels of depressive symptoms. Of the indirect effects, the strongest predictive effect is related to the pathway between academic stress and depression via resilience (at 78.64% of the indirect effects, effect size 0.081). The results confirm a serial mediation model for all HE students in predicting depressive symptoms from academic stress, resilience, and HE support. As the corresponding coefficients demonstrate contrasting directions, the analysis points towards a suppressive role of both resilience and HE support, as was proposed in the model hypothesis. As such, a 10% rise in perceived academic stress is linked to a 4.08–5.40% increase in depression symptom severity, but, through partial mediation of resilience and HE support, the former effect is suppressed by 2.13–3.03%. R^2^ indicates that the model predicts 32% of the variance, which is an adequate and substantial model fit (73, 74).

**Table 2:** Descriptive statistics and Pearson's correlation for all students on academic stress, depression, resilience, and HE support measures (*N* = 2,480).

		**M**	**SD**	**1**	**2**	**3**	**4**
1.	Academic Stress	3.371	0.854	(-)	0.404^***^	−0.379^***^	−0.282^***^
2.	Depression	2.280	0.619	.	(-)	−0.238^***^	−0.482^***^
3.	HE Support	2.950	0.895	.	.	(-)	0.149^***^
4.	Resilience	2.952	0.755	.	.	.	(-)

*** *significant at p < 0.001*.

### Low Academic Stress Students

When estimating the model effects for students who perceive low levels of academic stress, the total predictive effect of the model was not significant (*p* = 0.108). The result revealed just two significant partial effects (see Tables 3, 4, Figure 2B). The first includes a predictive effect of resilience on depression symptoms, with a coefficient of −0.281. For every unit increase in resilience, student depression symptoms were predicted to decrease 2.55–11.50%. The partial predictive effect of resilience on identification of HE support was significant at a threshold of 0.05, with a coefficient of 0.305. This finding suggests that higher resilience also predicts a higher capacity to identify HE support facilities.

**Table 3:** Descriptive statistics of the student subgroups on academic stress, depression, resilience, and HE support measures.

	**M**	**SD**
**Low academic stress (*****N*** **= 63)**		
Academic stress	1.480	0.289
Depression	1.762	0.568
HE Support	3.670	0.823
Resilience	3.476	0.794
**High academic stress (*****N*** **= 839)**		
Academic stress	4.611	0.283
Depression	2.557	0.602
HE Support	2.570	0.890
Resilience	2.708	0.746
**Depressed (*****N*** **= 404)**		
Academic stress	4.209	0.701
Depression	3.277	0.264
HE Support	2.660	0.953
Resilience	2.400	0.710
**Non-depressed (*****N*** **= 2,076)**		
Academic stress	3.619	0.848
Depression	2.085	0.461
HE support	3.010	0.872
Resilience	3.059	0.716

**Table 4:** Regression coefficients and significance tests for the explanatory model pathways between the five student groups.

**Pathway**	**b**	**t**	**p**	**R**	**95% CI**
**All students**				0.32	
a1	−0.250	−14.642	<0.001^***^		−0.283 to −0.216
a2	−0.383	−18.904	<0.001^***^		−0.423 to −0.344
b1	−0.324	−22.823	<0.001^***^		−0.352 to −0.297
b2	−0.055	−4.431	<0.001^***^		−0.080 to −0.031
d	0.054	2.352	0.019^*^		0.009 to 0.099
c′	0.190	14.114	<0.001^***^		0.163 to 0.216
X on Y	**Effect**	**se**	**t**	**p**	**95% CI**
Total	0.293	0.013	21.961	<0.001^***^	0.266 to 0.319
Ind. total	0.103	0.009			0.085 to 0.121
Ind1 (a1 x b1)	0.081	0.007			0.069 to 0.095
Ind2 (a2 x b2)	0.021	0.005			0.011 to 0.032
Ind3 (a1 x d x b2)	0.0007	0.0004			0.0001 to 0.0016
	**b**	**t**	**p**	**R**	**95% CI**
**Low academic stress**				0.19	
a1	−0.530	−1.534	0.130		−1.222 to 0.169
a2	−0.452	1.290	0.202		−1.152 to 0.249
b1	−0.281	3.144	0.003^***^		−0.460 to−0.102
b2	0.010	0.110	0.913		−0.164 to 0.183
d	0.305	2.399	0.020^*^		0.051 to 0.560
c′	0.259	1.086	0.282		−0.218 to 0.736
X on Y	**Effect**	**se**	**t**	**p**	**95% CI**
Total	0.402	0.247	1.632	0.108	−0.091 to 0.896
Ind. total	0.143	0.119			−0.070 to 0.398
Ind1 (a1 x b1)	0.149	0.105			−0.058 to 0.363
Ind2 (a2 x b2)	−0.004	0.049			−0.094 to 0.115
Ind3 (a1 x d x b2)	−0.0015	0.019			−0.045 to 0.039
	**b**	**t**	**p**	**R**	**95% CI**
**High academic stress**				0.19	
a1	−0.308	−3.406	<0.001^***^		−0.486 to −0.131
a2	−0.499	−4.621	<0.001^***^		−0.711 to −0.287
b1	−0.308	−12.145	<0.001^***^		−0.358 to −0.258
b2	−0.041	−1.910	0.057		−0.083 to 0.001
d	0.056	1.378	0.169		−0.024 to 0.137
c′	0.308	4.543	<0.001^***^		0.175 to 0.441
X on Y	**Effect**	**se**	**t**	**p**	**95% CI**
Total	0.424	0.072	5.872	<0.001^***^	0.282 to 0.565
Ind. total	0.116	0.033			0.055 to 0.183
Ind1 (a1 x b1)	0.095	0.029			0.039 to 0.154
Ind2 (a2 x b2)	0.020	0.012			−0.001 to 0.048
Ind3 (a1 x d x b2)	0.0007	0.001			−0.0003 to 0.0027
	**b**	**t**	**p**	**R**	**95% CI**
**Depressed**				0.05	
a1	−0.176	−3.531	<0.001^***^		−0.274 to−0.078
a2	−0.433	−6.640	<0.001^***^		−0.561 to−0.305
b1	−0.061	−3.302	0.001^**^		−0.097 to−0.025
b2	−0.039	−2.712	0.007^**^		−0.067 to−0.011
d	0.036	0.557	0.578		−0.091 to 0.162
c′	0.009	0.482	0.630		−0.029 to 0.048
X on Y	**Effect**	**se**	**t**	**p**	**95% CI**
Total	0.037	0.019	1.980	0.048^*^	0.0003 to 0.0739
Ind. total	0.028	0.009			0.011 to 0.046
Ind1 (a1 x b1)	0.011	0.005			0.003 to 0.022
Ind2 (a2 x b2)	0.017	0.008			0.003 to 0.033
Ind3 (a1 x d x b2)	0.0002	0.001			−0.0008 to 0.0015
	**b**	**t**	**p**	**R**	**95% CI**
**Non-depressed**				0.24	
a1	−0.191	−10.568	<0.001^***^		−0.226 to −0.156
a2	−0.369	−17.091	<0.001^***^		−0.411 to −0.326
b1	−0.221	−17.456	<0.001^***^		−0.246 to −0.196
b2	−0.041	−3.730	<0.001^***^		−0.062 to −0.019
d	0.043	1.675	0.094		−0.007 to 0.093
c′	0.133	11.618	<0.001^***^		0.110 to 0.155
X on Y	**Effect**	**se**	**t**	**p**	**95% CI**
Total	0.190	0.011	16.992	<0.001^***^	0.168 to 0.212
Ind. total	0.058	0.007			0.045 to 0.071
Ind1 (a1 x b1)	0.042	0.005			0.033 to 0.052
Ind2 (a2 x b2)	0.015	0.004			0.007 to 0.023
Ind3 (a1 x d x b2)	0.0003	0.0002			−0.0001 to 0.0008

*a1: independent variable (IV) to mediator 1; a2: IV to mediator 2; b1: mediator 1 to dependent variable (DV); b2: mediator 2 to DV; d: mediator 1 to mediator 2; c′: IV to dependent variable*.

* *significant at p < 0.05*;

*** *significant at p < 0.001*.

### High Academic Stress Students

The model effects for students who perceive high levels of academic stress, displayed a total predictive effect of.424 between perceived academic stress and depression (see Tables 3, 4, Figure 2C). For every unit increase in academic stress, depression levels rise 5.64–11.30%. Of this effect, 72.64% was related to the direct effect between academic stress and depression, with the remainder predominantly caused by the indirect effect via resilience. The protective effect of resilience predicts a 0.98–3.85% decrease in depressive symptoms. The predictive effect of resilience on HE support identification, and the predictive effect of HE support on depression remained insignificant. Results do not support a serial mediation model, but instead propose a single partial mediation model, where resilience partially mediates the relationship between academic stress and depression by acting as a suppressor. For the entire model, R^2^ is 0.19, which may be interpretated as an adequate and moderate model fit (73, 74).

### Depressed Students

Result from the model analysis for depressed students, yielded a low total predictive effect, with an effect size of 0.037 (see Tables 3, 4, Figure 2D). In addition, the model explained 5.41% of the variance present in the sample, thus demonstrating a lack of model fit for depressed students. Of the pathways tested to estimate serial mediation effects between academic stress and depression, results indicated no significant direct effect between academic stress and depression, and an insignificant serial effect between resilience and HE support identification. As such, results support complete mediation effects of both resilience and HE support, which implies a parallel mediation model.

The predictive effect of perceived academic stress on depression was predominantly mediated by HE support (60.71% of the indirect effect). However, effect sizes were small. A unit increase in resilience predicts a decrease in depressive symptoms of 0.63– or 2.43%, and a unit increase in HE support identification predicts a decrease in depression of 0.28–1.68%. In practise, the total effects of these mediating pathways predict a drop in mean depression scores ranging between 3.268 and 3.239. Scores thus remain within the range indicating presence of depression (61).

### Non-depressed Students

The total predictive effect of the model to assess depression levels in non-depressed HE students was 0.190, with a direct effect size of 0.133 (see Tables 3, 4, Figure 2E). This result indicates that 70.00% of the model's predictive effect originates from the direct predictive effect of academic stress on depressive symptoms. Both an indirect predictive effect via resilience and HE support proved significant, but the serial mediating pathway did not yield a significant result (effect size 95% Confidence Interval (CI) = −0.0001, −0.0008). The indirect effect was predominantly driven by the pathway of resilience mediation, at 72.41% of the total indirect effect. Results support independent partial mediating effects of resilience and HE support, where they act as suppressors of the relationship between academic stress and depression in parallel mediation. The largest effect originates from a direct predictive effect of academic stress on depression, at an estimated effect size of 0.133. A unit increase in perceived academic stress predicts an increase in depressive symptoms of 2.75–3.88%. Indirect effects collectively decrease depressive symptoms 1.13–1.78%.

## Discussion

The current study examined the relationships between Covid-19 related academic stress and depression with mediation effects of resilience and HE support, among students studying at higher education institutes during the corona crisis in the Netherlands. For all student subgroups, predictive suppressive effects of resilience on depression rates were demonstrated. More so, students subgroups experiencing low academic stress, and those experiencing depression, cease to demonstrate a direct effect of academic stress on predicted depression levels, whereas this direct effect is found for other student groups. In addition, a protective predictive effect through identification of HE support was significant for the entire student sample, as well as for subgroups of depressed, and non-depressed students, but not for low and high academic stress groups. Furthermore, serial mediation was demonstrated for all HE students in general, but it ceased to exist in subgroups. The study of these dynamics provides relevant insights as subgroup examinations were conducted based on research recommendations (16, 18). By comparing student groups based on levels of stress and depression, significant nuances and differences appear which give direction to strategies for student wellbeing enhancement at HE institutes.

In keeping with other studies on student wellbeing, the current study demonstrates a significant relationship between academic stress and depression among students (18, 75). When analysing the total sample, both resilience and HE support mediate the effect of academic stress on development of depressive symptoms for HE students, including a serial mediation between resilience and HE support.

A similar model is presented for non-depressed HE students, where approximately one third of the effect of academic stress on depression is mediated though resilience and HE support, though this group lacked serial mediation. Our findings support previous demonstrations of a protective mediating role for resilience and identification of support resources in the development of psychological problems among students (5, 28, 52), and provides corroboration for a link between resilience and the ability to identify helpful resources in the environment for students generally (59, 60).

For HE students experiencing low academic stress, higher resilience predicts lower depression scores, in addition to predicting higher identification of HE support resources. In contrast to other groups, students who experience low academic stress demonstrate no direct or indirect effect of academic stress on wellbeing due to the educational transition. A plausible explanation for this finding regards the higher resilience levels present among this subgroup, as higher resilience promotes higher levels of adaptive behaviours, in turn reducing negative impacts from perceived stress (58). More so, this subgroup may perceive the Covid-19 related educational transition as non-threatening, which also serves to protect against negative stress effects on student wellbeing (19).

When students do perceive high levels of academic stress during the Covid-19 crisis, the strongest predictive effect originates from a direct effect of academic stress on depression, and a predictive effect of resilience was found with the greatest indirect mediation. In contrast to the entire sample and depressed or non-depressed subgroups, the protective effect of HE support is lost for students with high levels of academic stress. This loss suggests a stress-induced impairment in students' ability to identify support facilities, which is supported by research describing inhibited adaptive behaviours if one's stress response becomes overwhelmed (54, 58). Akin to most student groups, students burdened by high academic stress stand to profit from resilience enhancement strategies and could benefit from programs focussed on the remediation of academic stress perception following the educational transition to online teaching.

As for HE students who experience depression, different outcomes emerge. Among depressed students, a relatively stronger mediation through HE support is found compared to the mediating effect of resilience. This finding indicates that the protective effects for this group are driven predominantly by the ability to identify HE support resources. Results may indicate that depressed students are turning to HE institutes in their search for support resources but remain unaware of their presence or are unsuccessful in locating available support facilities. Alternatively, students enduring psychological problems may experience help-seeking barriers, including the perception that no one will be able to offer the support that they need, which may negatively influence their ability to identify useful resources (24, 25). These students thus stand to benefit from promotion of comprehensive support facilities, a suggestion which has also been made in other studies on student wellbeing during the Covid-19 pandemic (43).

The lack of a strong protective mediation from resilience among depressed students reiterates previous work describing insufficient levels of resilience when psychopathology sets in (54, 58). The current findings suggest that depressed students require a different approach when forming strategies to increase wellbeing. This topic thus requires further attention in future research. For depressed students, the current explanatory model explains little variance, suggesting alternative pathways by which depression would be better predicted. With research demonstrating that 75% of people experiencing depression will have an onset before 24 years of age (11), it seems plausible that the depression rates captured regard recurrence or persistence and are thus not predicted by academic stress caused by the educational transition following the pandemic.

Instead, researchers propose that personality traits, comorbidity, or risk factors relating to hopelessness, and problem-solving capacity are all predictive of depression development (12, 75, 76). The supporting role of educational institutes in facilitating support for this burdened group of students should thus be subject of further investigation, especially given evidence that increased psychological distress is implicated in academic failure and study discontinuation (7, 8, 18).

### Limitations

Although the current study furthers understanding of the relationship between academic stress, resilience, and HE support on depressive symptoms for HE students during the Covid-19 pandemic in the Netherlands, there are some limitations. First, the sample contained an overrepresentation of female students. Although higher response rates from female students are often present within examinations of student populations (77), replications with balanced gender groups may provide added insight or nuances. Second, the cross-sectional nature of the current study would be enriched by examining student groups within a longitudinal design, where repeated measures study could examine the temporal persistence of Covid-19 related impacts on wellbeing. Third, with little variation captured for depressed HE students, this group should receive independent focus to identify relevant explanatory pathways, as well as to reveal potential avenues for HE support and intervention. Fourth, this study assessed academic stress *via* four relevant stressors, although additional sources of academic stress are found among students, including time constraints, parental pressures, teachers' expectations, and self-perceptions (78, 79). As such, further study including additional sources of academic stress will serve to improve understanding of the collective and independent effects of academic stressors on student wellbeing. Finally, with resilience scores generally within the lower range in the current student sample, additional study of resilience among HE students will expand collective knowledge and serve to further inform enhancement strategies.

### Practical Implications and Future Directions

According to our findings, the greatest overall improvements to HE student wellbeing during the Covid-19 pandemic can be attained by promoting resilience in addition to decreasing perceived Covid-19 related academic stress for specific subgroups. Higher educational institutes should focus on student perceptions of academic workload, expectations, and anticipated study delays, and how to remedy stress elevations which hamper psychological wellbeing through resilient response. More so, expanding perceived academic stress measures will aid research on students' academic stress experiences beyond the confines of the pandemic, as research demonstrates that students also experience academic stressors in non-pandemic academic settings (7, 78). Furthermore, an exploration of means with which to increase resilience should yield fruitful wellbeing enhancement strategies. The Covid-19 related restrictions that preclude live contact, need not act as a barrier for proactive development of tools that promote resilience among students during this time, as online and informal resources can also offer benefits to wellbeing (24, 25, 47, 48).

With studies proposing that informal support is generally preferred by young adults due to financial considerations, higher availability, and lower associations with stigmatisation (25), their applicability within the currents predictive models deserves further scrutiny. Moreover, research indicates that facilitators of help-seeking among students include increased education and awareness, encouragement, removal of treatment scepticism, and the provision of accessible resources such as student counselling (23, 24, 27). As such, HE institutes could stimulate student wellbeing by exploring relevant facilitators of student support seeking, in addition to scrutinising HE support service accessibility and availability.

The current findings also argue for a differential research approach when examining wellbeing of HE students who are experiencing depression. These students may not receive any notable benefit from perceived academic stress reduction or resilience enhancement, and as such require further research to identify relevant predictors and effective interventions. It may also be the case that this group requires support services that are not typically available *via* HE institutes, or that a lack of academic attendance resulting from psychological distress keeps these students outside of the range of HE support services. Given their difficult disposition, understanding wellbeing dynamics of depressed students warrants continued exploration.

The current study offers HE institutes in the Netherlands enriched understanding on how to best support student wellbeing throughout the remainder of the Covid-19 pandemic, based on group levels of academic stress and depressive symptoms, which had not been investigated previously. If future circumstances demand student isolation, students may continually be required to conduct studies via an online educational environment for extensive periods of time. Under such circumstances, mitigation of perceived academic stress and enhancement of resilience offer protective means with which to positively promote student wellbeing.

## Conclusions

For HE students studying in the Netherlands, model testing demonstrates that perceived academic stress positively predicts depressive symptoms during the coronavirus pandemic and its implications for online education. Moreover, within the model test for the entire student sample, protective serial mediation is present *via* resilience, and HE support. Subgroup examinations demonstrated parallel mediation, partial predictive effects, in addition to a lack of model fit for specific subgroups of students. These findings suggest that HE institutes may increase student wellbeing generally by enhancing resilience and HE support, as well as by decreasing perceived academic stress. However, specific approaches could be required if the aim concerns enhancement of student wellbeing among student subgroups. Wellbeing enhancement among students during of the Covid-19 pandemic should be strategically reviewed by HE institutes and should include focus on support service availability, visibility, and range of services.

## Data Availability Statement

The datasets presented in this article are not readily available because participants of this study did not consent to public availability of their data. Upon request from collaborating researchers within the C19 ISWS consortium, data is available as relevant consent was provided by participants. Requests to access the datasets should be directed to Rutger Kappe, rutger.kappe@inholland.nl.

## Ethics Statement

The studies involving human participants were reviewed and approved by Ethics Committee for Social Sciences and Humanities at the University of Antwerp and the Institutional Review Board InHolland University of Applied Sciences. Written informed consent for participation was not required for this study in accordance with the national legislation and the institutional requirements.

## Author Contributions

RK aided in survey design, survey distribution, and contribution of materials. MV analyzed the data and wrote the manuscript. RK and MV conducted literature research. Both authors contributed to the article and approved the submitted version.

## Funding

This work was supported by the department of Student Success, InHolland University of Applied Sciences, Haarlem, the Netherlands.

## Conflict of Interest

The authors declare that the research was conducted in the absence of any commercial or financial relationships that could be construed as a potential conflict of interest.

## Publisher's Note

All claims expressed in this article are solely those of the authors and do not necessarily represent those of their affiliated organizations, or those of the publisher, the editors and the reviewers. Any product that may be evaluated in this article, or claim that may be made by its manufacturer, is not guaranteed or endorsed by the publisher.

## Appendix

**Table A1:** Government response stringency Index for the Netherlands with implementation dates of government-imposed restrictions.

**Date**	**Government response stringency index**	**Description**
February 27^th^, 2020	5.56	First national case of Covid-19 in Tilburg (Northern Brabant).
March 6^th^, 2020	11.11	First Covid-19 related death and subsequent first implementations of covid-19 related health advice for Brabant province.
March 13^th^, 2020	53.70	Introduction of flight restrictions from high-risk countries, restriction on group sizes in all public domains and a ban on teaching at location for all higher educational institutes
March 23^rd^, 2020	78.70	Start of the “Intelligent Lockdown”.
May 11^th^, 2020	71.30	First ease of government-imposed restrictions, including the reopening of public libraries, middle schools, and some outdoor sport activities.

**Table A2:** Covariate analysis.

**Scale**		**Sociodemographic variables**			
		**Gender**	**Age**	**Migration status**	**HE Generation**
Depression	*Pearson's r*	0.074	0.101	0.081	0.045
	*p-value*	<0.001^***^	<0.001^***^	<0.001^***^	0.027^*^
Aca stress	*Pearson's r*	0.009	−0.012	0.007	0.068
	*p-value*	0.664	0.550	0.721	0.001^***^
Resilience	*Pearson's r*	−0.159	−0.041	0.015	−0.041
	*p-value*	<0.001^***^	0.042^*^	0.466	0.043^*^
HE Support	*Pearson's r*	0.037	−0.073	−0.046	−0.036
	*p-value*	0.067	<0.001^***^	0.023^*^	0.072

*A significant independent direct effect on depression was taken as reason to include the sociodemographic variable as a covariate in the final analysis (N = 2,480)*.

* *significant at p < 0.05*;

*** *significant at p < 0.001*.
